# Electrochemical Processing and Thermal Properties of Functional Core/Multi-Shell ZnAl/Ni/NiP Microparticles

**DOI:** 10.3390/ma14040834

**Published:** 2021-02-09

**Authors:** David Svetlizky, Honorata Kazimierczak, Bar Ovadia, Ariel Sharoni, Noam Eliaz

**Affiliations:** Department of Materials Science and Engineering, Tel-Aviv University, Ramat Aviv, Tel-Aviv 6997801, Israel; dsvetlizky@gmail.com (D.S.); honorata.kazimierczak@gmail.com (H.K.); ovdbar@gmail.com (B.O.); ariel879@gmail.com (A.S.)

**Keywords:** galvanic displacement, autocatalytic deposition, electroless deposition, coating, zinc alloy, core-shell powder, thermal stability, additive manufacturing, phase change materials (PCMs)

## Abstract

Electroless deposition on zinc and its alloys is challenging because of the negative standard potential of zinc, the formation of poor surface layers during oxidation in aqueous solutions, and extensive hydrogen evolution. Therefore, there are only few reports of electroless deposition on Zn and its alloys, neither of them on micro/nano powders. Here, we propose a two-step process that allows the formation of compact, uniform, and conformal Ni/NiP shell on Zn-based alloy microparticles without agglomeration. The process utilizes controlled galvanic displacement of Ni deposition in ethanol-based bath, followed by NiP autocatalytic deposition in an alkaline aqueous solution. The mechanism and effect of deposition conditions on the shell formation are discussed. Thermal stability and functional analysis of core-shell powder reveal a thermal storage capability of 98.5% with an encapsulation ratio of 66.5%. No significant morphological change of the core-shell powder and no apparent leakage of the ZnAl alloy through the Ni shell are evident following differential scanning calorimetry tests. Our two-step process paves the way to utilize electroless deposition for depositing metallic-based functional coatings on Zn-based bulk and powder materials.

## 1. Introduction

Functional metal/metal core-shell powders/particles attract great interest due to their unique characteristics and multi-functionality. The specific combination of chemical compositions of the core and shell practically defines the potential fabrication techniques and the resulting core-shell functionality [1,2]. The outer shell may be used to protect the core material in various ways: (1) Thermal barrier at elevated temperatures [3]; (2) physical barrier which provides a proper encapsulation of the core (in both solid and liquid states) [4]; (3) diffusion barrier which is designed to inhibit elemental segregation or interdiffusion between the core material and any material in its surrounding [5]; and (4) reflective/absorptive surface, e.g., to either reduce or increase laser absorbance in the metal powder during additive manufacturing (AM) [6,7]. In addition, the functionality of the outer shell may be beneficial when it reacts with its surrounding to: (1) Enhance metallurgical bonding with the matrix in a composite material [8,9]; (2) promote specific desired reaction [10]; and (3) enhance properties of the end material, e.g., corrosion resistance [11], increased re-melting temperature [12], density, or mechanical properties.

Various applications of metal/metal core-shell materials have been explored, such as core-shell Sn-Cu anodes for Li rechargeable batteries [13], surface modification of Zn-based electrode (made of Zn granulated powder) in zinc-air batteries [14], enhancement of powder metallurgy processability [15,16,17], AM applications [9,10,18,19,20], phase change materials (PCMs) for thermal energy storage [3,21,22], die-attach materials with high remelting temperature [4,12,23,24,25], corrosion resistance enhancement of Fe-based coating processed by plasma spraying [11], sensors [26,27], and catalysis [28] applications. The selection of the encapsulation process is highly dependent on the selected core and shell chemistries, shell thickness, the final quantity of coated powder, and the overall desired functionality.

Zn and its alloys are characterized by high flowability, low melting temperatures, fair mechanical properties, and good processability by forming and plating [29]. Such properties make Zn and its alloys in general, and Zn-based powders specifically, attractive for various applications, e.g., sacrificial anodes in cathodic protection or die-castings. However, these materials are usually limited to non-structural applications as they tend to suffer from degradation in mechanical properties due to aging even at room temperature (RT) [29]. Recent studies proposed Zn-based alloys/powders as PCMs for thermal energy storage applications [21,30,31,32,33,34,35], due to their unique thermophysical properties such as adequate phase change temperature, high heat capacity, low thermal expansion, high heat fusion, and high thermal conductivity [32], compared to conventional PCMs such as inorganic salts, paraffin, and fatty acids [30]. The proper encapsulation of PCMs is vital to provide adequate heat transfer control, which allows a volume change of the PCM during melting-solidification cycling [21]. Furthermore, the encapsulation of the PCMs is important to ensure a large specific surface area during the cyclic heating-cooling process for efficient thermal energy transfer [35]. It is important to note that the fabricated encapsulation shell material should provide sufficient ability to encapsulate the PCM material for numerous thermal cycles without any leakage of the core material [35,36].

Ni has been proposed as a potential encapsulation material for Zn-based PCMs [21,22,35] or Zn-based anodes in zinc-air batteries [14,37]. Ni and its alloys offer good wear, corrosion, and chemical resistance properties [38]. Furthermore, Ni is characterized by a high melting point (~1455 °C) and provides oxidation resistance up to about 500 °C. These make Ni an adequate material for high-temperature applications in general [38], and a good candidate specifically for surface modification of zinc powders. Ni coating produced via electroplating has been reported to be an adequate encapsulation of macro-sized Pb platelets in PCM for waste heat recovery applications [39]. Ni was also used both as an encapsulation shell of Cu PCM and as a catalyst of methane steam reforming at the PCM's melting temperature (1084 °C) [40].

Zn and Zn-based alloy surfaces could perhaps be coated with Ni using various techniques such as chemical vapor deposition (CVD), atomic layer deposition (ALD), intermittent electrodeposition, barrel plating, and electroless deposition. CVD [41] and ALD [42], for example, have been employed to coat Zn with silica and titania, respectively, for battery anodes. Compared to the fluidized bed-based techniques, the electrochemical-based methods such as intermittent electrodeposition and electroless deposition are relatively straightforward, low-cost, and allow for the precise control of the process and coating properties. However, intermittent electrodeposition on particles was reported to result in both a substantial agglomeration of the coated powders and high surface roughness of the coating [43,44].

In electroless plating, contrary to electrodeposition, coupled electrode reactions take place without the application of an external voltage or current. Hence, electroless plating can be applied to substrates that cannot be connected to a current source, e.g., individual powder particles, thus forming core-shell materials [45,46]. Electroless deposition is also simple, cost effective, and uses simple and cheap equipment. The deposited coating is often uniform, compact, and conformal, which enables coating of geometrically complex surfaces [47]. Electroless deposition is categorized into two major processes: (1) Autocatalytic deposition, in which a reducing agent present in the electrolyte solution is being utilized to supply electrons to facilitate the reduction reaction of metal ions to the substrate surface. Autocatalytic deposition can be applied to non-conductive materials by applying surface activation pretreatment before the deposition step [45,46,48,49], and (2) galvanic displacement deposition, also known as cementation [50,51,52,53], in which the displacement reaction does not require the presence of any reducing agent in the electrolyte solution to facilitate deposition. This process occurs spontaneously when a less noble metal is immersed in an electrolyte solution containing more noble metal ions. The galvanic displacement process takes place until the substrate is fully covered with the depositing metal, thus terminating the oxidation-reduction reaction. Consequently, the final coating thickness is limited [45,48,52,54,55].

Electroless deposition of Ni-based coatings has been extensively studied and applied in a variety of industrial applications [56,57]. Many reports have explored electroless deposition of Ni-based coatings on chemically reactive materials such as Mg- and Al-based alloys. It was shown to be challenging, and sometimes impossible, to achieve high-quality coating when surface pretreatments were not applied due to the simultaneous occurrence of substrate oxidation (dissolution) and coating during the deposition process [58,59,60,61,62,63,64].

Electroplating and electroless deposition of chemically/electrochemically active materials such as Zn, Mg, and Al are highly challenging when an aqueous-based solution is used as an electrolyte [64,65]. Zn has a fairly negative standard electrode potential; it would act as an anode in the galvanic cell created when it is in contact with more noble metals in electrolyte solutions. In addition, its oxidation reaction in aqueous media results in the formation of a non-uniform and porous oxide/hydroxide layer over the core surface [64], and might also be accompanied by extensive hydrogen evolution. This could raise hydrogen safety concerns when depositing large batches of powder due to the inherently high surface area of the powder. Given these phenomena, it is not surprising that there are only few reports of electroless deposition on Zn and its alloys, none of them on powders. To the best of the author’s knowledge, only one (not peer reviewed) article has reported a procedure for Ni plating on Zn-based alloy [66]. In that report, a five-step electroless Ni plating process was conducted, alternatingly, in mild alkaline and acidic electrolyte solutions, on Zn-based parts manufactured by die casting. However, this process is applicable mainly to relatively large items, because inevitable chemical dissolution of the Zn-based alloy surface and galvanic displacement with Ni take place during the process. Such a process is not useful for coating of small items such as micro/nano powders, because of their high surface area and rapid surface dissolution accompanied by extensive hydrogen evolution. Alloying Zn with even more active metals such as Al or Mg would make these undesirable reactions more pronounced [65].

Here, we propose a two-step electroless deposition process, subsequently in anhydrous and aqueous media, for the encapsulation of Zn-based microparticles by Ni/NiP shell [67]. Our goal is to develop a practical process for the deposition of continuous, conformal, metallic layers without degrading the zinc core powder. The effects of deposition conditions on the shell formation are studied by chemical, microstructural, and thermal characterization.

## 2. Materials and Methods

### 2.1. Powder Feedstock and Material Characterization

The two-step deposition process was carried out on a prealloyed powder feedstock, processed via a gas atomization process. The powder was synthesized by TLS Technik GmbH & Co. Spezialpulver KG (Bitterfeld-Wolfen, Germany) especially for this study, with a target composition of Zn92Al8 (wt.%), known as ZA-8. The obtained particle size distribution was between 45 and 150 μm, having in mind applications such as directed energy deposition (DED) additive manufacturing. This powder is referred herein as the ZnAl core powder. In general, Zn-Al alloys are zinc casting alloys suitable to applications requiring high as-cast strength, hardness, and wear resistance. Inductively coupled plasma optical emission spectrometry (ICP-OES, PlasmaQuant PQ9000, Analytik Jena AG, Jena, Germany) was utilized to determine the chemical composition of the as-received feedstock powder (Table 1).

The morphology of the powder particles and their local chemical composition were characterized before and after encapsulation using a scanning electron microscope (SEM, Quanta 200 FEG, FEI, Waltham, MA, USA) equipped with an energy-dispersive X-ray spectrometer (EDS, INCA detector, Oxford Instruments, Abington, UK). Samples for cross-section analysis were prepared by cold mounting the powder particles in acrylic resin (Struers, Copenhagen, Denmark), followed by mechanical grinding on 1200, 2500, and 4000 grit SiC papers to expose the core-shell cross-section. Final mechanical polishing was done using 1 µm of diamond suspension.

X-ray diffractometer (D8 ADVANCE, Bruker AXS, Madison, WI, USA) equipped with a Cu-Kα radiation source was used to characterize the crystallography of the powder before and after encapsulation. Phase identification and crystallographic orientation were analyzed using TOPAS software ver. 5 (Bruker AXS, Madison, WI, USA). The thermal stability and functionality of the core-shell material were determined using differential scanning calorimetry (DSC) (Q20, TA Instruments, New Castle, DE, USA), followed by morphological characterization in SEM. In the DSC tests, the powder was placed in an alumina crucible under a nitrogen environment, heated to 470 °C, and cooled down to 80 °C at a rate of 2 °C/min.

### 2.2. Two-Step Core-Shell Encapsulation Process

The first deposition step (cementation) formed a thin layer of Ni on the as-received powder, whereas the second deposition step (autocatalytic deposition) formed NiP on the outer shell on top of the predeposited Ni layer. Each time, a suspension of 20 g/dm^3^ of powder was introduced into the deposition bath. The suspension solution was kept homogenously stirred using mechanical stirrer to allow uniform coating of individual particles without agglomeration. Temperature-controlled glycerol bath maintained the bath solution at the desired temperature. After each step, the coated powders were washed thoroughly with ethanol absolute/distilled water, followed by drying at 70 °C in ambient conditions.

#### 2.2.1. Step #1—Ni Cementation

The chemical composition of the electrolyte solutions used in the two-step core-shell formation process and the corresponding operating conditions are given in Table 2. Here, solution 1 and solution 2 are referred to as the cementation and autocatalytic solutions, respectively. The cementation solution was prepared by dissolving nickel chloride (the source of Ni ions) in ethanol absolute (the solvent). Vanillin was used as a surfactant which enhances surface wettability by reducing the surface energy of the bath solution, thus promoting and improving the deposition quality [68].

To identify proper conditions for the cementation process, various process conditions were evaluated. First, the effect of deposition temperature was studied (55–60, 65–70, and 70–75 °C) at a constant solution composition of 0.1 M NiCl_2_·6H_2_O (Alpha Aesar, Haverhill, MA, USA) and process duration of 15 min. Then, the effect of nickel chloride concentration was evaluated (0.05, 0.10, 0.20, and 0.30 M), while keeping the concentration of additive (1 g/L vanillin), deposition temperature (65–70 °C), and deposition duration (15 min) constant. Finally, the effect of deposition time was studied (15, 30, and 60 min), while keeping the concentration of nickel chloride (0.20 M), the concentration of vanillin (2 g/L), and deposition temperature range (65–70 °C) constant.

#### 2.2.2. Step #2—NiP Autocatalytic Deposition

Once the Ni/ZnAl core-shell powder was formed via a cementation process, a second, top shell of NiP was deposited using an autocatalytic deposition process. The electrolyte solution was prepared by dissolving nickel sulfate (Alpha Aesar, Haverhill, MA, USA) (the source of Ni ions) in DI water (18.2 MΩ/cm). Sodium citrate (Na_3_C_6_H_5_O_7_·2H_2_O) (Alpha Aesar) was used as a buffer and a complexing agent. Sodium hydroxide (Alpha Aesar) was used to adjust the pH. The deposition process was evaluated at various deposition times (25, 35, and 75 min).

## 3. Results and Discussion

### 3.1. Introduction of the Challenge: One-Step NiP Autocatalytic Deposition on ZnAl Powder

**Figure 1:**
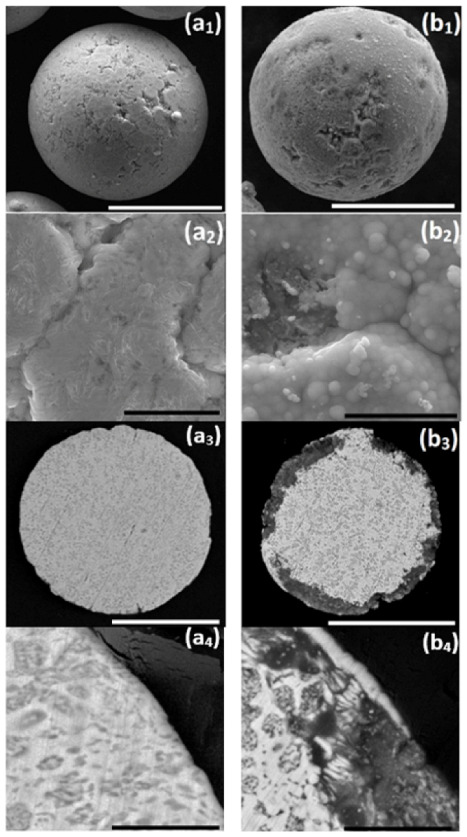
One-step autocatalytic NiP deposition (**b1**–**b4**) on untreated ZnAl powder (**a1**–**a4**). Deposition was done at *T* = 45 °C for *t* = 25 min. (**a1**,**a2**,**b1**,**b2**) Secondary electron (SE) images of the powder surface and (**a3**,**a4**,**b3**,**b4**) backscatter electron (BSE) images of the powder cross-section. White scale bars equal 50 μm, black scale bars equal 5 μm.

**Figure 2:**
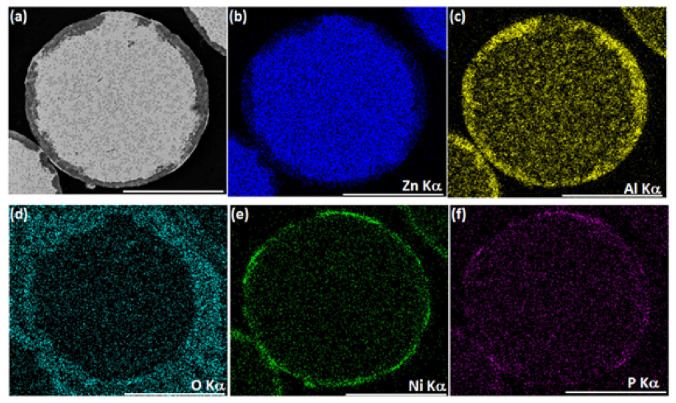
One-step autocatalytic NiP deposition (*T* = 45 °C, *t* = 25 min) on untreated ZnAl powder. SEM-BSE image of a typical ZnAl powder cross-section after NiP deposition (**a**) and the corresponding EDS elemental maps of Zn, Al, O, Ni, and P. Scale bars equal 50 μm (**b**–**f**).

First, a conventional, one-step, autocatalytic NiP deposition from an aqueous electrolyte solution on ZnAl powder was studied. No pretreatment step was applied to the powder in this case. The as-received ZnAl alloy powder was introduced into a mechanically stirred aqueous plating solution (see Table 2) at 45 °C. When ZnAl powder is immersed in an electrolyte solution containing Ni^2+^ ions (Table 2, solution 2), galvanic displacement reaction is inevitable due to a large electrode potential difference between zinc and aluminum compared to nickel (*E*^0^_Zn_^2+^_/Zn_ = −0.762 V, *E*^0^_Al_^3+^_/Al_ = −1.662 V, *E*^0^_Ni_^2+^_/Ni_ = −0.257 V vs. SHE). Figure 1 shows the ZnAl powder before and after one-step autocatalytic NiP deposition trial. It is evident that the dissolution of zinc and aluminum occurred intensively as a result of a galvanic displacement process. The dissolution of the ZnAl powder coincides and competes with the desirable autocatalytic NiP deposition processes, thus preventing the formation of a continuous, high-quality NiP layer on the surface of the particles. EDS elemental analysis of the cross-section of the powder after the one-step process (Figure 2) shows that a small amount of nickel is deposited on the surface of the ZnAl particles, but not in the form of a uniform and conformal layer (Figure 2e). Moreover, while zinc is directly dissolved in the electrolyte solution (Figure 2b), aluminum tends to segregate on the surface, forming a relatively thick non-uniform oxide layer (Figure 2c,d). The observed phenomena can be described by the following reactions [69,70]:

Galvanic displacement, anodic:

(1) $$\mathrm{Zn}\to {\mathrm{Zn}}^{2+}+2e^{-}\;\;\;\;\;\;\;\;\;\;E^{0}\;=\;-0.762\;V$$

(2) $$\mathrm{Al}\to {\mathrm{Al}}^{3+}+3e^{-}\;\;\;\;\;\;\;\;\;\;E^{0}\;=\;-1.662\;V$$

Galvanic displacement, cathodic:

(3) $${\mathrm{Ni}}^{2+}+2e^{-}\to \mathrm{Ni}\;\;\;\;\;\;\;\;\;\;E^{0}\;=\;-0.257\;V$$

In addition, in aerated aqueous solutions, both hydrogen and oxygen depolarization (Equations (4) and (5)) may, in principal, take place:

(4) $$2H_{2}O+2e^{-}\to H_{2}+2{\mathrm{OH}}^{-}\;\;\;\;\;\;\;\;\;\;E^{0}\;=\;-0.828\;V$$

(5) $$2H_{2}O+O_{2}+4e^{-}\to 4{\mathrm{OH}}^{-}\;\;\;\;\;\;\;\;\;\;E^{0}\;=\;+0.401\;V$$

Furthermore, the products of the anodic (Equation (2)) and cathodic (Equations (4) and (5)) processes can easily react with each other, resulting in the formation of aluminum hydroxide (Equation (6)), which converts into oxide (Equation (7)):

(6) $$Al^{3+}+3{\mathrm{OH}}^{-}\to Al{\left(OH\right)}_{3}$$

(7) $$2Al{\left(OH\right)}_{3}\to {\mathrm{Al}}_{2}O_{3}+3H_{2}O$$

Even if Equations (4) and (5) do not occur, the excess of ${\mathrm{OH}}^{-}$ naturally present in alkaline solutions can be the reagent in Equation (6). The aluminum phase present in the core particle might as well react with ${\mathrm{OH}}^{-}$, forming aluminum hydroxide or hydroxyaluminate [69]:

(8) $$Al+3{\mathrm{OH}}^{-}\to Al{\left(OH\right)}_{3}+3e^{-}\;\;\;\;\;\;\;\;\;\;E^{0}\;=\;-2.31\;V$$

(9) $$Al+4{\mathrm{OH}}^{-}\to Al{\left(OH\right)}_{4}^{-}+3e^{-}\;\;\;\;\;\;\;\;\;\;E^{0}\;=\;-2.33\;V$$

Next, this may lead to the formation of aluminum oxides and hydroxides on the coated substrate [71]. Furthermore, when solution 2 (see Table 2) is used, the autocatalytic NiP deposition is accompanied by the anodic reaction of hypophosphite oxidation [70]:

(10) $$H_{2}{\mathrm{PO}}_{2}^{-}+H_{2}O\to H_{2}{\mathrm{PO}}_{3}^{-}+2H^{+}+2e^{-}\;\;\;\;\;\;\;\;\;\;E^{0}\;=\;-0.504\;V$$

The anodic reaction is coupled with nickel deposition (Equation (3)), possible hydrogen and oxygen depolarization processes (Equations (4) and (5)), as well as with hypophosphite ion reduction to phosphorus [70]:

(11) $$H_{2}{\mathrm{PO}}_{2}^{-}+2H^{+}+e^{-}\to 2H_{2}O+P\;\;\;\;\;\;\;\;\;\;E^{0}\;=\;-0.391\;V$$

The lack of compact and thick metallic layers on the powder particles after the one-step autocatalytic deposition process, together with the relatively poor detectability of phosphorus (Figure 2f), indicates that the autocatalytic NiP deposition is significantly hindered in the applied conditions. It can be presumed that nickel reduction occurs mainly as a result of galvanic displacement, coupled with the oxidation of zinc and aluminum (Equations (1) and (2)). Hydrogen evolution (Equation (4)), which accompanies these processes, was confirmed by the observation of intensive gas bubbles in the solution during the process, resulting in partial blocking of the powder surface during the plating process. This also significantly hampers the formation of a continuous metallic shell on the ZnAl powder. Such an intensive hydrogen evolution reaction indirectly promotes the above-mentioned aluminum oxide formation (Equations (6) and (7)) by producing a relatively large excess of ${\mathrm{OH}}^{-}$. Moreover, the processes of zinc and aluminum dissolution change the bath chemical composition, while intensive hydrogen co-evolution might also have an impact on the bath pH (especially in the zone close to the particle/solution interphase), which also hinders the process of autocatalytic NiP deposition.

We also tested other aqueous electrolyte solutions for NiP autocatalytic deposition on ZnAl powder. Unfortunately, all of the studied NiP plating baths (pH = 5–10, *T* = 45–90 °C, with the use of various complexing agents [72,73,74,75]) gave similar results, yielding low-quality NiP deposits on the thick surface oxide layer. SEM-EDS revealed in all cases Ni-based deposits only partially covering the particles, along with thick and brittle oxide layer, often with some cracks and coating delamination. In conclusion, the results in Section 3.1 clearly demonstrate the poor outcomes obtained when employing a conventional, one-step, autocatalytic NiP deposition from an aqueous electrolyte solution on ZnAl powder. Such process is not suitable for forming uniform and continuous Ni-based encapsulation of ZnAl microparticles.

### 3.2. First-Step Cementation in Ethanol

To overcome the challenges presented above, the use of an organic solvent was introduced. Ethanol was chosen because its hydroxyl group has a polar nature, it easily dissolves many ionic compounds, and is considered an environmentally friendly solvent [76]. Moreover, it was claimed that the presence of ethanol in electrolyte solutions can suppress the anodic dissolution of Zn and Al and the formation of its oxides on the immersed metal surfaces [77,78]. It has also been noted that the presence of aluminum ions interfere with nickel cementation in nickel sulfate aqueous solutions [79]. In this step, no reducing agents were added to the electrolyte solution. The process of simple galvanic displacement in the NiCl_2_-ethanol solution (solution 1, Table 2) was investigated as a method for Ni coating on ZnAl powder. The optimization process of the cementation pretreatment was based on experimental optimization of the main factors influencing the cementation process: (1) Process temperature, (2) NiCl_2_ concentration, and (3) process duration.

#### 3.2.1. The Effect of Temperature

**Figure 3:**
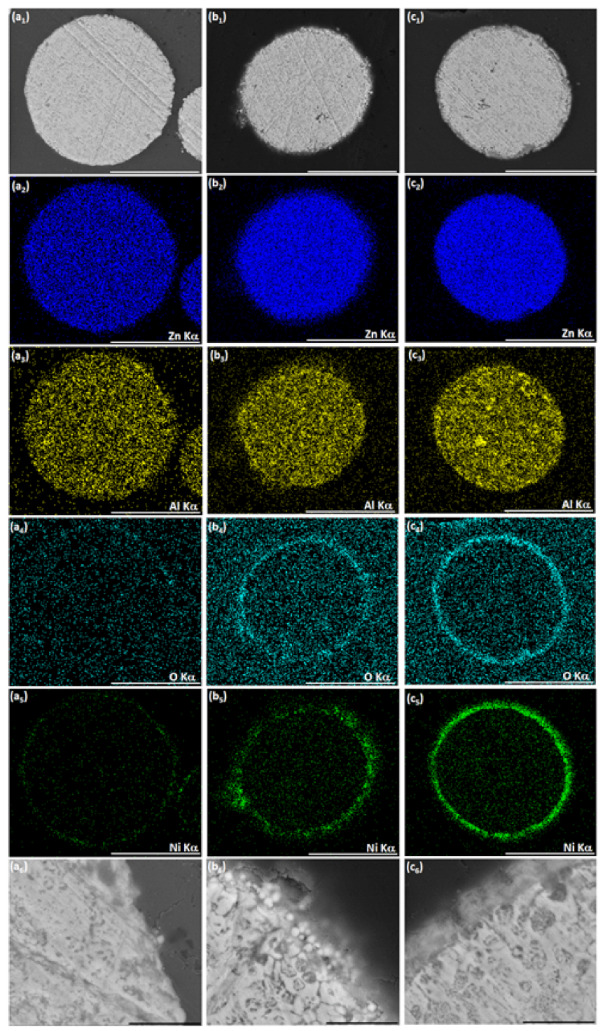
The effect of bath temperature on the cementation of Ni on ZnAl powder in ethanol-based bath. (**a1**–**a6**) *T* = 55–60 °C, (**b1**–**b6**) *T* = 65–70 °C, and (**c1**–**c6**) *T* = 70–75 °C. SEM-BSE images of a particle cross-section and the corresponding EDS elemental maps of Zn, Al, O, and Ni. White scale bars equal 50 μm, black scale bars equal 5 μm.

The effect of bath temperature on the Ni cementation on ZnAl microparticles was tested in the 0.10 M NiCl_2_-ethanol system. The ZnAl powder was introduced into the cementation solution (solution 1, Table 2) for 15 min, in different temperature regimes: *T*_1_ = 55–60 °C, *T*_2_ = 65–70 °C, and *T*_3_ = 70–75 °C (Figure 3). At temperatures lower than 55 °C, no reaction occurred. On the other hand, bath temperature could not exceed 75 °C to avoid boiling and fast evaporation of ethanol.

The process in the lowest temperature regime studied (Figure 3(a1–a6)) led to the formation of only a low amount of Ni on the particle surface. Comparing the high-magnification image of this particle’s cross-section (Figure 3(a6)) to that of an untreated particle (Figure 3(a4)), no noticeable metallic layer was produced in the ethanol-based bath at *T* = 55–60 °C. The increase of temperature to 65–70 °C resulted in the deposition of Ni in the form of spherical particles (with diameter between ca. 100 to 500 nm) on the ZnAl powder surface. These particles look brighter in the scanning electron microscope-backscatter electrons (SEM-BSE) image (Figure 3(b5,b6)), indicating metallic Ni deposition. The increased amount of oxygen on the surface of the particles (Figure 3(b4)) and the slightly darker areas between Ni crystallites on the layer formed on the ZnAl powder (Figure 3(b6)) indicate that some nickel oxide might have also been formed. The highest bath temperature (Figure 3(c1–c6)) led to the formation of more compact and uniform layer which, however, consisted entirely of nickel oxide. Nevertheless, it should be emphasized that no severe dissolution of either Zn or Al took place, and that no aluminum oxide formed in the ethanol-based process, regardless of bath temperature. The temperature range *T* = 65–70 °C was identified as most adequate for further investigation.

#### 3.2.2. The Effect of NiCl_2_ Concentration

**Figure 4:**
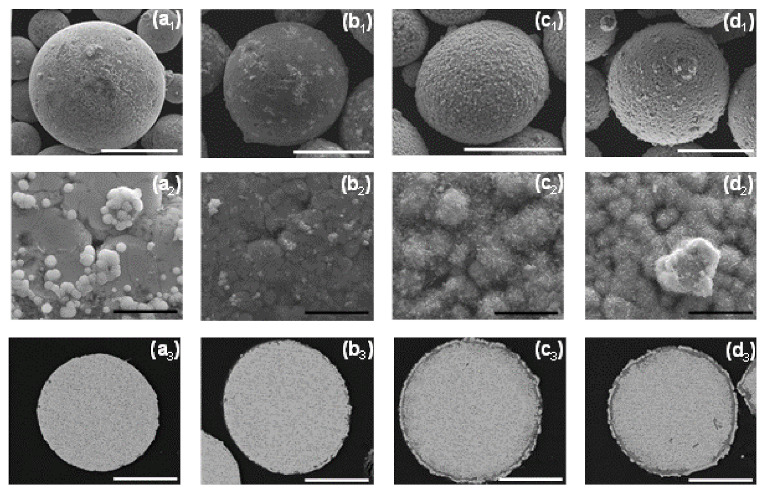
Cementation of Ni in ethanol-based bath on ZnAl powder at *T* = 65–70 °C. (**a1**–**a3**) 0.05 M, (**b1**–**b3**) 0.10 M, (**c1**–**c3**) 0.20 M, and (**d1**–**d3**) 0.30 M NiCl_2_. Top row: SEM-BSE images of the cross-section, middle, and bottom rows: SE images of particles’ surfaces. White scale bars equal 50 μm, black scale bars equal 5 μm.

The quality of the Ni deposit can be easily controlled by the nickel chloride concentration in the ethanol-based solution (Figure 4). The increase of NiCl_2_ concentration, from 0.05 M to 0.20 M, results in a significant change in the morphology of the deposit—from Ni crystallites of different sizes scattered on the ZnAl particles (Figure 4(a2,b2)) to a metallic Ni layer covering the whole particle, relatively uniformly (Figure 4(c1–c3)). The darker areas below the Ni layer (Figure 4(c3)) indicate some oxide formation. EDS analysis (not shown herein) revealed this material to be aluminum oxide. Nevertheless, particle dissolution and oxide formation were much slower in the ethanol-based solution (Figure 3 and Figure 4) than in an aqueous electrolyte (Figure 1 and Figure 2). The Ni deposit obtained by such a cementation process in ethanol established a slightly porous encapsulation layer on ZnAl particles (Figure 4(c1–c3)). Further increase of the nickel chloride concentration in the bath resulted in a more developed surface morphology of the Ni encapsulation layer with protruding particles, which might be more difficult to continuously coat in further processes. A thicker oxide layer also formed below the Ni deposit, especially in areas below pores in the coating (Figure 4(d3)).

#### 3.2.3. The Effect of Process Time

**Figure 5:**
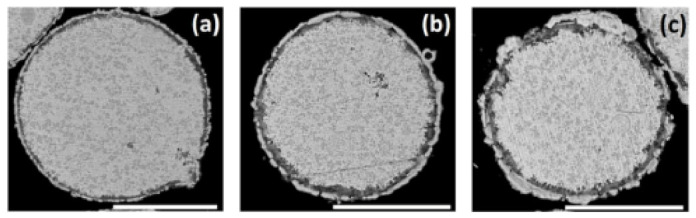
Cementation of Ni on ZnAl alloy particles in ethanol-based bath. SEM-BSE images of the cross-sections of particles in solution 2 (Table 2), treated for (**a**) 15, (**b**) 30, and (**c**) 60 min. Scale bars equal 50 μm.

Longer process times in the ethanol-based solution yielded a thicker Ni deposit on ZnAl particles (Figure 5). However, the reduction of Ni (Equation (3)) on a large cathodic surface area is coupled with a significantly higher rate of oxidation reactions. This results in much smaller anodic areas defined by holes and discontinuities in the Ni deposit. When Ni deposit is formed on the majority of the surface of the particles, further Ni reduction occurs on the cathodic sites on the surface of the core particles. This is also coupled with oxidation processes that take place in the pores and cracks of the deposited Ni. This phenomenon can be described as pitting corrosion of ZnAl particles, in which intensive Zn and Al dissolution (Equations (1) and (2)) may be accompanied by hydrogen evolution (Equation (4)). Some H_2_O molecules may be introduced to the ethanol-based solution together with nickel chloride, according to the oxygen reduction reaction (Equation (5)). Then, an oxide layer is formed according to Equations (6) and (7). All of these hinder the uniform growth of a Ni layer on the particle’s surface. Furthermore, the intensive corrosion of ZnAl under the Ni deposit causes shell delamination from the particles’ surfaces. This phenomenon prevents the system from reaching equilibrium. Hence, the longer the duration of the process, the more developed and discontinuous the Ni deposit formed is, and the larger the part of corroding ZnAl particles, forming a brittle and thick oxide layer.

To summarize this part, the conditions found in this work for Ni deposition by galvanic displacement in an ethanol-based solution allow the formation of relatively compact, uniform, and conformal metallic shell. However, some pores in the coating could not be avoided. Therefore, one cannot expect such a Ni coating to serve alone as the ultimate encapsulation of the ZnAl particles. Based on the results obtained in Section 3.2, the optimal galvanic displacement conditions were defined and were used for all further investigations discussed in Section 3.3 (*T* = 65–70 °C, 0.20 M NiCl_2_, *t* = 15 min).

### 3.3. Second-Step Encapsulation of ZnAl/Ni Particles

**Figure 6:**
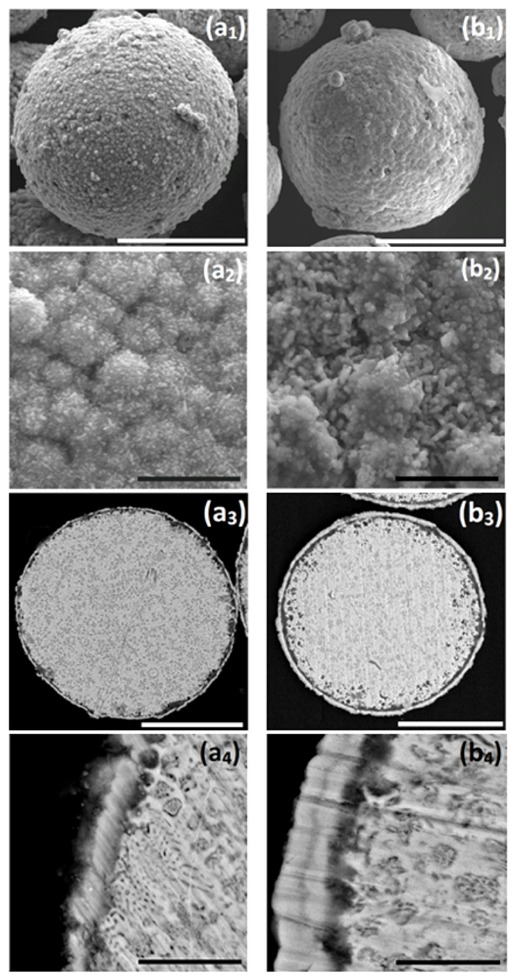
(**a1**,**a2**,**b1**,**b2**) SEM-secondary electrons (SE) images of surface morphology, (**a3**,**a4**,**b3**,**b4**) SEM-BSE images of cross-sections of: (**a1**–**a4**) ZnAl particle after the optimized Ni cementation process, and (**b1**–**b4**) ZnAl particle after Ni cementation process followed by autocatalytic NiP deposition. Cementation: 0.20 M NiCl_2_, *T* = 65–70 °C, *t* = 15 min, 1.5 g/L vanillin; autocatalytic deposition: 0.14 M Cit, 0.12 M NiSO_4_, 0.40 M NaH_2_PO_2_, pH = 9, *T* = 45–50 °C, *t* = 25 min. White scale bars equal 50 μm, black scale bars equal 5 μm.

**Figure 7:**
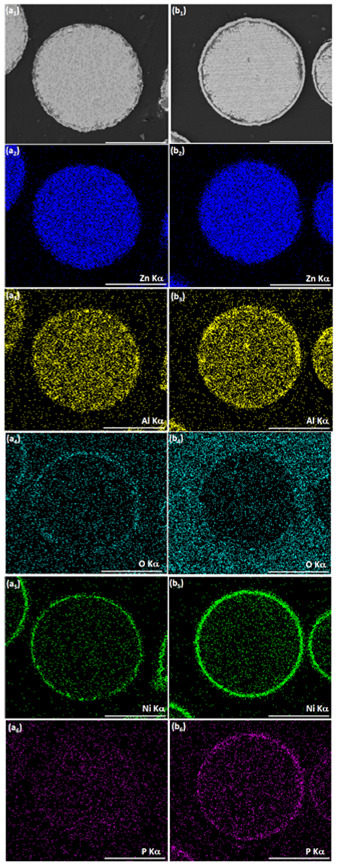
SEM-BSE images of cross-sections of (**a1**) ZnAl particle after optimized Ni cementation process, (**b1**) ZnAl particle after optimized Ni cementation process followed by autocatalytic NiP deposition. (**a2**–**a6**, **b2**–**b6**) Corresponding EDS elemental maps of Zn, Al, O, Ni, and P. Scale bar equals 50 μm.

When ZnAl powder deposited with Ni by galvanic displacement in ethanol (Figure 4(c1–c3), Figure 6(a1–a4), and Figure 7(a1–a6)) is introduced into an aqueous plating bath (solution 2, Table 2), autocatalytic deposition of NiP occurs without the problems described before. Comparing the surface morphology and the cross-section of ZnAl particles after one-step Ni deposition (Figure 6(a1–a4)) to those after the two-step Ni/NiP deposition process (Figure 6(b1–b4)), it is evident that the top shell is continuous, and that no significant dissolution of the ZnAl core powder occurred. Elemental mapping (Figure 7) confirms Ni and P codeposition (Figure 7(b5,b6)) on the first Ni layer (Figure 7(a5,a6)). Some oxide layer is also formed under the Ni/NiP coating (Figure 7(b3)); however, it does not affect the quality and adhesion of the deposited metallic shell on the ZnAl core. No agglomeration of the encapsulated microparticles was observed following the two-step deposition process.

**Figure 8:**
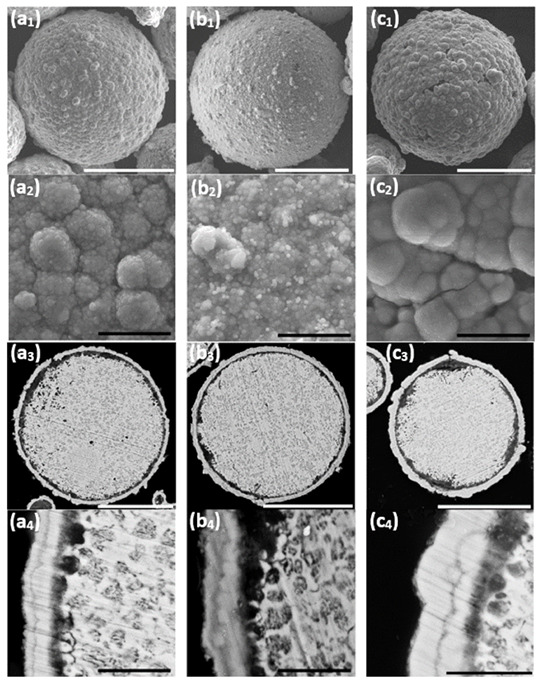
The effect of duration of the autocatalytic deposition time. (**a1,a2**,**b1,b2**,**c1,c2**) SEM-SE images revealing the particles’ surface morphology, (**a3**,**a4**,**b3**,**b4**,**c3,c4**) SEM-BSE images of cross-sections of ZnAl particles coated with Ni/NiP via Ni cementation followed by autocatalytic NiP deposition. (**a1**–**a4**) 25 min, (**b1**–**b4**) 35 min, and (**c1**–**c4**) 75 min. White scale bars equal 50 μm, black scale bars equal 5 μm.

It is thus shown that while the first Ni layer deposited during the cementation process is not fully continuous and contains some pores and holes, it does function as a barrier layer, substantially slowing down galvanic displacement processes during the autocatalytic deposition process, which could lead to either corrosion of the ZnAl core (Equations (1)–(3)) or formation of an oxide layer (Equations (6) and (7)). Furthermore, the first Ni layer acts as a catalyst for the hypophosphite ions oxidation (Equation (10)) that accompanies the reduction of Ni and P (Equations (3) and (11)). The autocatalytic NiP deposition proceeds continuously on the first Ni layer, and the thickness of the second (NiP) layer can be controlled directly by the deposition time (Figure 8).

Longer immersion time of the powders in the aqueous plating solution carries a greater risk of pitting corrosion of the ZnAl alloy (Figure 8(c3)). This leads to the formation of cracks in the Ni/NiP coating and to a thicker oxide layer (Figure 8(c2,c3)).

A 25 min duration of the autocatalytic deposition process was found adequate for the formation of good quality (i.e., continuous, crack-free, uniform, and conformal) NiP coatings on top of the first Ni shell, without significant changes of the ZnAl core or noticeable cracking of the metallic Ni shell.

It is important to note that the two-step process presented herein is not limited to the chemistry used in this work, but provides numerous degrees of freedom for selecting the desired material chemistry of the shell according to the application and depending on the conventional electroless depositable materials. Furthermore, although this study was carried out on Zn-based alloy powder, the process is also applicable on bulk substrates (e.g., rods, plates, and sheets) made of Zn and its alloys.

### 3.4. Characterization of the ZnAl/Ni/NiP Core-Shell Powder

After the two-step deposition process, no agglomeration of the micro-encapsulated particles was observed. The average shell thickness obtained via the two-step deposition process of the ZnAl alloy microparticles was measured by image analysis, yielding a shell thickness of 2.0 ± 0.6 μm (based on 50 measurements on 10 randomly selected encapsulated microparticles at different positions in the cross-section). The bi-shell layer thickness can be controlled by either adding NiP autocatalytic deposition steps or changing the number of ZnAl microparticles in solution. Moreover, since the bi-shell reported herein is uniform, compact, and conformal, it is possible to apply on top of it any established metal/alloy/composite autocatalytic deposition process with no additional pretreatments, thus forming a multilayer core-shell material according to the desired functionality.

**Figure 9:**
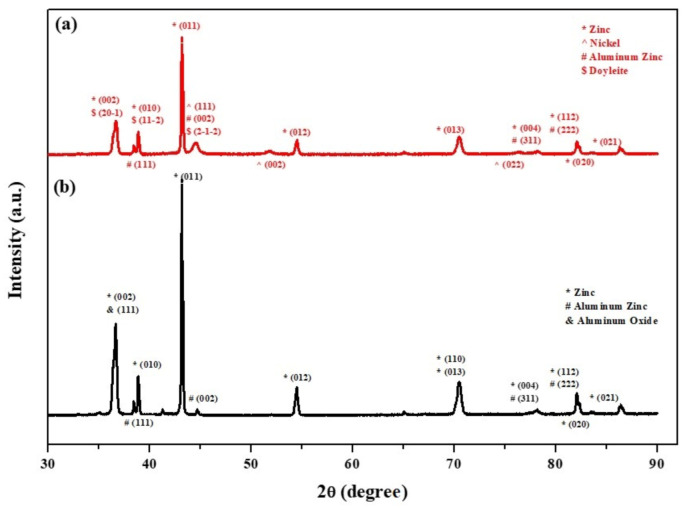
XRD patterns from: (**a**) ZnAl core powder coated with a Ni layer via cementation process and a second, top NiP layer via an autocatalytic process, and (**b**) untreated ZnAl powder.

Phase identification of the core-shell particles before and after the two-step deposition process was done by XRD analysis. Figure 9b shows the XRD pattern of the core powder in the untreated state. The ZnAl alloy particles consist mainly of a Zn phase (ICDD card No. 04-003-5661; hcp, *a* = 2.6710 Å, *c* = 4.8960 Å), with Al-rich solid solution phase Al_0.52_Zn_0.48_, with the numbers representing the atomic fraction (ICDD card No. 04-020-7570; fcc, *a* = 4.0520 Å). This analysis is in accordance with the microstructure shown in Figure 1(a4) and with the microstructure of a similar alloy (ZA-8) consisting of Al-rich dendrites in a matrix of Zn-rich eutectic phase [80,81]. Furthermore, native Al_2_O_3_ (ICDD card No. 00-056-0456; *a* = 11.9694 Å, *b* = 2.9472 Å, *c* = 5.5693 Å) is present on the surface of the untreated ZnAl alloy particles. Figure 9a shows the XRD pattern from core-shell powders after the two-step process. Substantial reduction in the intensity of reflections from the core powder is evident, indicating the presence of a coating on top of the ZnAl core powder. The XRD pattern from the encapsulated core-shell microparticles consists of both the Zn and Al-rich reflections that originated from the core material (at the same angles as in the pattern of the untreated powder), with an additional reflection corresponding with the strongest (111) reflection of the Ni phase (ICDD card No. 04-010-6148, fcc, *a* = 3.5136 Å). This confirms the formation of metallic Ni on top of the ZnAl core particles, in agreement with the EDS elemental maps. The XRD pattern in Figure 9a also reveals the presence of Doyleite, an Al(OH)_3_ phase (card No. 04-009-2200; triclinic, *a* = 5.0942 Å, *b* = 5.3276 Å, *c* = 4.9931 Å). The formation of Al(OH)_3_ can be explained by the excess of ${\mathrm{OH}}^{-}$ naturally present in the alkaline solution used for the autocatalytic process, forming aluminum hydroxide according to Equation (8).

The functionality of the core-shell powder is defined by the chemical, physical, and thermal properties of both the core and shell materials. For example, the Ni-based shell acts as a physical, thermal, and diffusion barrier at elevated temperatures. Such a Ni encapsulation may also provide enhanced heat transfer for thermal energy storage and PCM applications due to its high thermal stability, thermal conductivity (compared to ceramic-based encapsulation such as alumina, silica, etc.), high contact surface area for heat transfer, and the ability to hold its structural integrity (during the melting and solidification of the core) [22,82]. The deposited Ni-shell may act as protective encapsulation in thermal energy storage based on latent heat storage applications [39,40]. For this purpose, a conformal, uniform, and pore-free core encapsulation is vital. Such an encapsulation shell must be conformal, uniform, and pore-free at the individual particle level, otherwise leakage of the core material, loss of structural integrity, or enhanced corrosion of the core might occur at elevated temperatures, during the solid-to-liquid phase transformation, and harm its functionality [82].

**Figure 10:**
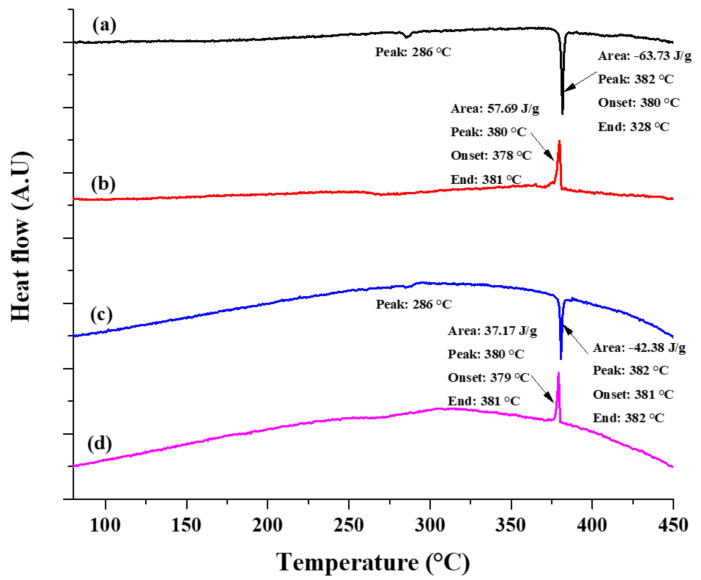
Differential scanning calorimetry (DSC) heating (**a**) and cooling (**b**) thermograms of untreated ZnAl microparticles, and heating (**c**) and cooling (**d**) thermograms of the core-shell microparticles prepared using the two-step process.

In this study, the thermal stability and functionality of the core-shell microparticles were determined using DSC analysis. The DSC thermograms of the heating and cooling phases of the untreated and encapsulated ZnAl/Ni core-shell microparticles are shown in Figure 10. All analyzed thermograms exhibit one main endothermic peak at 382 °C. This peak is ascribed to the latent heat generated during the solid-to-liquid phase transformation, corresponding to the melting temperature of the ZnAl alloy core [3]. Comparison of the enthalpy of the two analyzed samples shows that while the corresponding temperatures of the endothermic peaks are similar for both samples, the heat capacity of the core-shell powder decreases by 33.5% compared to the untreated core powder. This decrease in the heat capacity is ascribed to the Ni-based encapsulation shell and to the oxide layer formed inherently during the two-step deposition process. Additional small endothermic peaks in the range of 282–289 °C can be attributed to the latent heat during the eutectic phase transformation of the ZnAl alloy core. Several characteristics of the core-shell material were calculated according to Equations (12) to (14) [83,84,85], and are summarized in Table 3.

(12) $$\mathrm{Encapsulation}\;\mathrm{ratio}\;=\;\;\frac{\Delta H_{m,\mathrm{core}-\mathrm{shell}}}{\Delta H_{m,\mathrm{core}}}\times 100\%$$

(13) $$\mathrm{Encapsulation}\;\mathrm{ratio}\;=\;\;\frac{\Delta H_{m,\mathrm{core}-\mathrm{shell}}}{\Delta H_{m,\mathrm{core}}}\times 100\%$$

(14) $$\mathrm{Thermal}\;\mathrm{energy}\;\mathrm{capability}\;=\;\;\frac{\mathrm{Encapsulation}\;\mathrm{ratio}}{\mathrm{Encapsulation}\;\mathrm{efficiency}}\times 100\%$$

where Δ*H*_m,core_ and Δ*H*_m,core-shell_ are the melting heat capacities, and Δ*H*_c,core_ and Δ*H*_c,core-shell_ are the solidification heat capacities of the core material and the core-shell material, respectively.

Both the encapsulation ratio and encapsulation efficiency measure the effectiveness of the obtained core-shell encapsulation. However, Equation (13) also considers the undercooling effect, which may occur during the solidification stage [83]. Slight undercooling can be observed in the DSC thermographs in Figure 10. The slight difference between the calculated encapsulation ratio and encapsulation efficiency also indicates that some undercooling occurs during the cooling stage. The thermal storage capability of the core-shell particles was also calculated (see Table 3) and these microparticles exhibit a thermal storage capability of 98.52%. This indicates that most of the analyzed core-shell microparticles exhibited effective latent heat storage and release during the solid-liquid-solid phase change processes.

**Figure 11:**
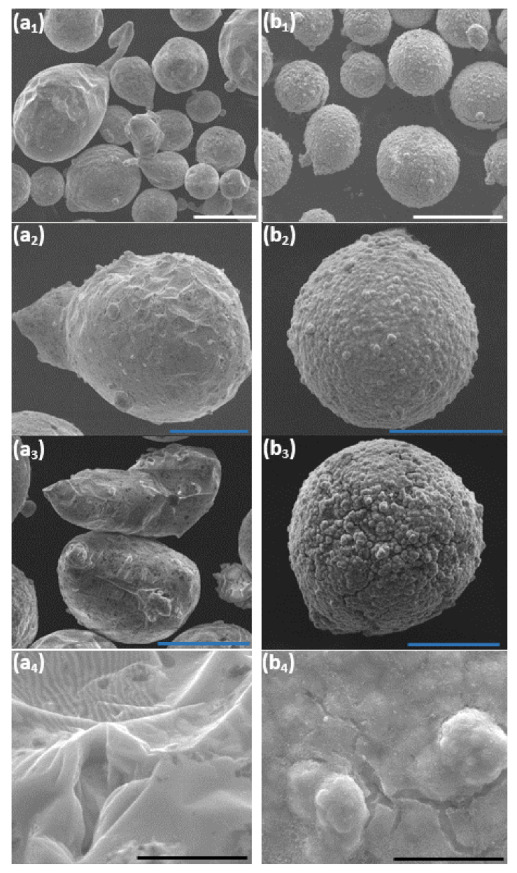
SEM images at various magnifications acquired after DSC testing up to 450 °C of untreated (**a1**–**a4**) and encapsulated (**b1**–**b4**) ZnAl particles. The scale bars in (**a1**,**b1**) equal 100 μm, in (**a2,a3**,**b2**,**b3**) equal 50 μm, and in (**a4**,**b4**) equal 5 μm.

Changes in the morphology and in the thermal stability of the untreated core and the encapsulated core-shell microparticles were studied. Both samples after DSC analysis were examined in the SEM. Comparison of the surface morphology of the untreated particles before (Figure 1(a1,a2)) and after (Figure 11(a1–a4)) thermal treatment (RT–450 °C) reveals a significant morphological change. The heat-treated core particles do not retain their structural integrity, resulting in geometrical shape changes of the core particles (from spherical to distorted shape). This is ascribed to the full melting of the ZnAl alloy core particles, which is evident in the DSC thermographs (Figure 10a,b). Figure 11(b1–b4) shows the morphology of the encapsulated core-shell microparticles a after DSC test. No significant morphological change is evident in this case. The analyzed core-shell particles retain their structural integrity, with no apparent substantial leakage of ZnAl alloy through the Ni shell, while the DSC thermographs (Figure 10c,d) clearly show that the encapsulated ZnAl alloy core was fully melted. Yet, in some of the core-shell particles, some cracking in the shell was observed, which can be ascribed to: (1) Different thermal expansion coefficients of the core and shell materials, and (2) volume expansion of the core material during the solid-liquid phase transformation [36].

## 4. Conclusions

In conclusion, a two-step electroless deposition process was developed for the encapsulation of ZnAl alloy microparticles with Ni/NiP shell. The effects of Ni and NiP deposition conditions on the quality of deposited encapsulation were discussed. The novel two-step process allowed the formation of a continuous, compact, and conformal metallic encapsulation shell on ZnAl alloy powder, with a shell thickness of 2.0 ± 0.6 μm. Good adhesion of the shell to the core particles is obtained, with no apparent agglomeration of the coated powder particles. It is shown that a conventional one-step electroless plating from an aqueous solution on the ZnAl powder could not be applied to obtain high-quality Ni-based encapsulation due to the high chemical reactivity of the core material. Ni deposited by galvanic displacement formed a thin metallic encapsulation which served as a barrier between the ZnAl alloy core and the aqueous electrolyte, thus preventing its intensive dissolution, and as a catalytic surface for the oxidation of the reducing agent in the second step. The microstructure of the encapsulation shell consisted of a mixture of pure metallic Ni and Al(OH)_3_. The ZnAl/Ni/NiP core-shell microparticles exhibited a thermal storage capability of 98.52%, encapsulation efficiency of 65.52%, and encapsulation ratio of 66.5%. They retained their structural integrity, with no apparent substantial leakage of ZnAl alloy through the Ni shell after DSC testing. The two-step deposition process is not limited to the coating chemistry or substrate geometry used in this study. The functionality of the ZnAl/Ni/NiP core-shell powder is yet to be evaluated in applications such as phase change materials (PCMs) for thermal energy storage, anodes in zinc-air batteries, or fabrication of novel metal matrix composites by additive manufacturing or spark plasma sintering (SPS).

## Figures and Tables

**Table 1:** Chemical composition of the gas-atomized ZnAl alloy powder feedstock, analyzed by inductively coupled plasma optical emission spectrometry (ICP-OES).

Element	Concentration, wt.%
Zn	91.024
Al	7.791
Cu	1.121
Mg	0.021
Fe	0.021
Sn	0.020
Pb	0.002

**Table 2:** Composition of the deposition solutions and deposition conditions (varied process parameters are in Italics).

Bath Composition and Deposition Conditions	Solution 1	Solution 2
NiSO_4_·6H_2_O (M)	-	0.12
Na_3_C_6_H_5_O_7_·2H_2_O (M)	-	0.14
NaH_2_PO_2_·H_2_O (M)	-	0.40
Solvent	Ethanol absolute	Deionized (DI) water
pH	-	9
Temperature (°C)	*55–60, 65–70,70–75*	45–50
NiCl_2_·6H_2_O (M)	*0.05, 0.1, 0.2, 0.3*	-
Time (min)	*15, 30, 60*	*25, 35, 75*

**Table 3:** Phase transformation characteristics of untreated versus encapsulated core-shell powder.

Sample	Δ*H*_m_ J/g	Δ*H*_c_ J/g	Encapsulation Ratio, %	Encapsulation Efficiency, %	Thermal Storage Capability, %
Core	63.73	57.69	N/A	N/A	N/A
Core-shell	42.38	37.17	66.50	65.52	98.52

## Data Availability

Data cannot be shared at this time since it is an ongoing R&D project.
